# Intrathoracic neurogenic tumors (ITNs): Management of solid and cystic lesions

**DOI:** 10.1111/1759-7714.14927

**Published:** 2023-05-18

**Authors:** Giovanni Natale, Stefano Forte, Gaetana Messina, Beatrice Leonardi, Rosa Mirra, Francesco Leone, Vincenzo Di Filippo, Davide Gerardo Pica, Francesca Capasso, Mary Bove, Antonio Noro, Giorgia Opromolla, Mario Martone, Sabrina De Angelis, Alfonso Fiorelli

**Affiliations:** ^1^ Department of Translation Medicine, Thoracic Surgery Unit Università della Campania “Luigi Vanvitelli” Naples Italy; ^2^ Genomics and Experimental Oncology Unit IOM Ricerca Viagrande Italy

**Keywords:** ITNs, neurogenic tumors, schwannoma, paravertebral tumors

## Abstract

**Background:**

Intrathoracic neurogenic tumors (INTs) are derived from nerve tissue and grow within the chest. Preoperative diagnosis can be challenging and only complete surgical exeresis enables confirmation of the suspected diagnosis. Here, we analyzed our experience on management of paravertebral lesions with solid and cystic patterns.

**Methods:**

A monocentric retrospective study was conducted, which included 25 consecutive cases of ITNs in the period from 2010 to 2022. These cases had been surgically treated by thoracoscopic resection alone, or in combination with neurosurgery in the case of dumbbell tumors. The demographic and operative data along with complications were recorded and analyzed.

**Results:**

Twenty‐five patients were diagnosed with a paravertebral lesion of which 19 (76%) had solid features and six (24%) had cystic features. The most common diagnosis was schwannoma (72%), followed by neurofibroma (20%) and malignant schwannoma (8%). In four cases (12%) the tumor showed an intraspinal extension. None of the patients had recurrence until 6 months of follow‐up. Comparison between the VATS and thoracotomy procedures showed that outcome of discharge on the postoperative day, on average, was 2.61 ± 0.5 versus 3.51 ± 0.53, respectively (*p*‐value <0.001).

**Conclusion:**

The treatment of choice for INTs is complete resection which is tailored to tumor size, location, and extension. In our study, paravertebral tumors with cystic characteristics were not associated with an intraspinal extension and did not show a different behavior from solid tumors.

## Graphical abstract

Intrathoracic neurogenic tumors (INTs) are derived from nerve tissue and grow within the chest. Preoperative diagnosis can be challenging and only complete exeresis allows to have the confirmation of the suspected diagnosis. Here, we analyzed our experience on management of paravertebral lesions with solid pattern and cystic pattern.

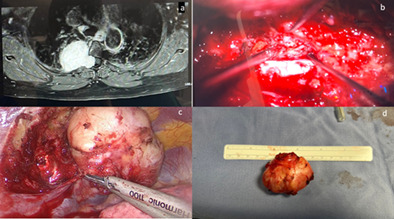

## INTRODUCTION

Intrathoracic neurogenic tumors (INTs) are derived from, or are from cells of the nerve sheaths, autonomic ganglia, or paraganglia and grow within the chest which are predominantly located in the paravertebral area and are defined as lesions of the posterior mediastinum.^1^

The histological classification of INTs is based on the nervous structure of origin. Tumors arising from nerves sheath are classified as schwannoma, neurofibroma, and malignant peripheral nerve sheath tumors (MPNSTs). Those arising from the sympathetic chain ganglion cells are classified as ganglioneuroma, ganglioneuroblastoma, and neuroblastoma, and those arising from the parasympathetic autonomic nervous system associated with the paraganglia are classified as paraganglioma.

Intrathoracic neurogenic tumors are uncommon and represent 15%–25% of mediastinal tumors, generally occurring in the posterior paravertebral area. They can also originate from the chest wall, large airways, and lungs. Schwannomas are the most common intrathoracic neurogenic tumors (INTs) in adults (20%) and usually arise as primitive lesions not associated with other pathologies, compared with neurofibromas that are mainly associated with syndromes such as neurofibromatosis 1 (NF1).^2^ A schwannoma appears as a well encapsulated tumor, firm, and grayish tan in color. Neoplastic cell proliferation occurs in the endoneurium, and the perineurium forms the capsule. It can have two different cellular patterns: Antoni type A with a dense avascular spindle‐cell pattern and Antoni type B in which myxomatous changes are seen associated with cystic areas, vascular thickening, and frequent areas of hemorrhage. No clinical differences in the behavior of two different cell patterns are described. It is a benign tumor which rarely degenerates into a malignant tumor. A neurofibroma is characterized by cells that have proliferated in a disorderly manner and is a pseudoencapsulated tumor. Malignant schwannoma invades locally and may have distant metastases. Other histotypes of ITN are extremely rare in adults.^3^ In this study, we report our experience in the management of both solid and cystic paravertebral masses suspected to be INTs and their surgical treatment.

## METHODS

A retrospective review of the medical records of 25 patients diagnosed with intrathoracic neurogenic tumors and treated with complete surgical excision in the Thoracic Surgery Unit of the University of Campania Luigi Vanvitelli between 2010 and 2022 was conducted.

The clinical and demographic parameters of the study population were evaluated and statistically analyzed. In this study, all the surgical records of the clinical cases collected evaluated the surgical approach used, operating times, intraoperative blood losses, postoperative stay days, and scale of the postoperative pain. Mortality, morbidity, and recurrence rate were assessed. All pathology specimens were examined by a pathologist experienced in the examination of soft tissue tumors. The study protocol was reviewed and approved by the Ethics Committee of the University. All patients signed an informed consent for the use of pathological specimens for scientific purposes.

Data are summarized as mean and standard deviation (SD) for continuous variables, and absolute number and percentage for categorical variables. Correlations were calculated using Pearson's coefficient. *p* < 0.05 was considered to be statistically significant. MedCalc statistical software (version 12.3) was used for the analysis.

### Preoperative assessment

**FIGURE 1:**
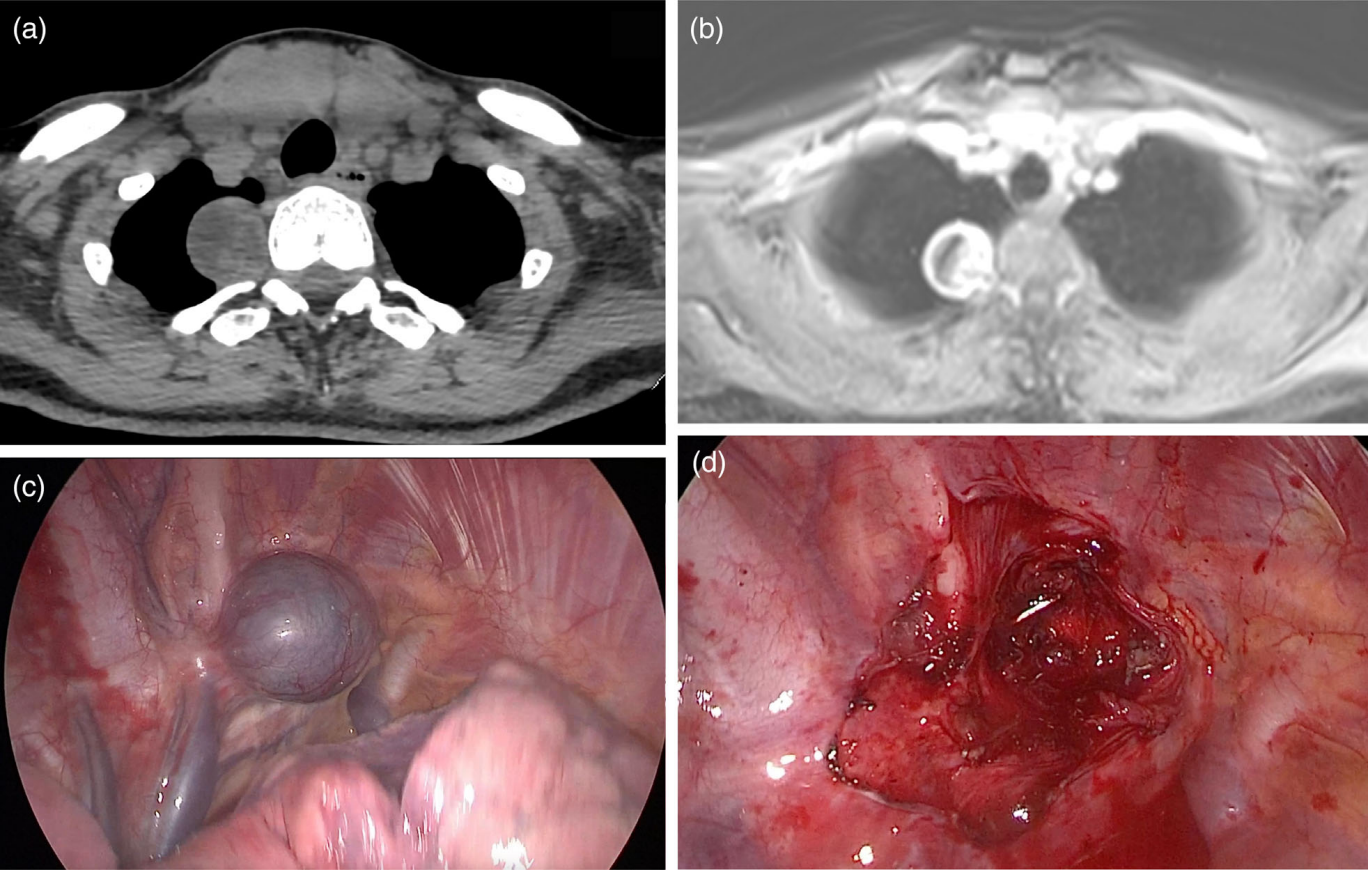
A 26‐year‐old woman with a symptomatic intrathoracic neurogenic tumor (ITN) on the right T2/3. Computed tomography (CT) scan and magnetic resonance imaging (MRI) did not show a spinal extension of the tumor (a, b) that was removed by right video‐assisted thoracoscopic surgery (VATS) (c, d).

Diagnosis was occasional, due to local mass effect or nerve dysfunction by a posterior mediastinal mass. The first evaluation was represented by a correct medical history and objective examination. The diagnosis of ITN preferably required a contrast‐enhanced computed tomography (CT) scan to identify the site of mass occurrence, as shown in Figure 1. In cases of suspected spinal extension, magnetic resonance imaging (MRI) was required. The radiological characteristics of the intrathoracic mass and the patient's clinical history led to a diagnosis of ITN confirmed by post‐surgical definitive histological reports. Surgical excision was the treatment of choice which was planned according to the assessment of the morphological characteristics of the tumor. Preoperative clinical evaluations for the anesthesiological evaluation were laboratory tests, electrocardiogram, and spirometry.

### Operative technique

**FIGURE 2:**
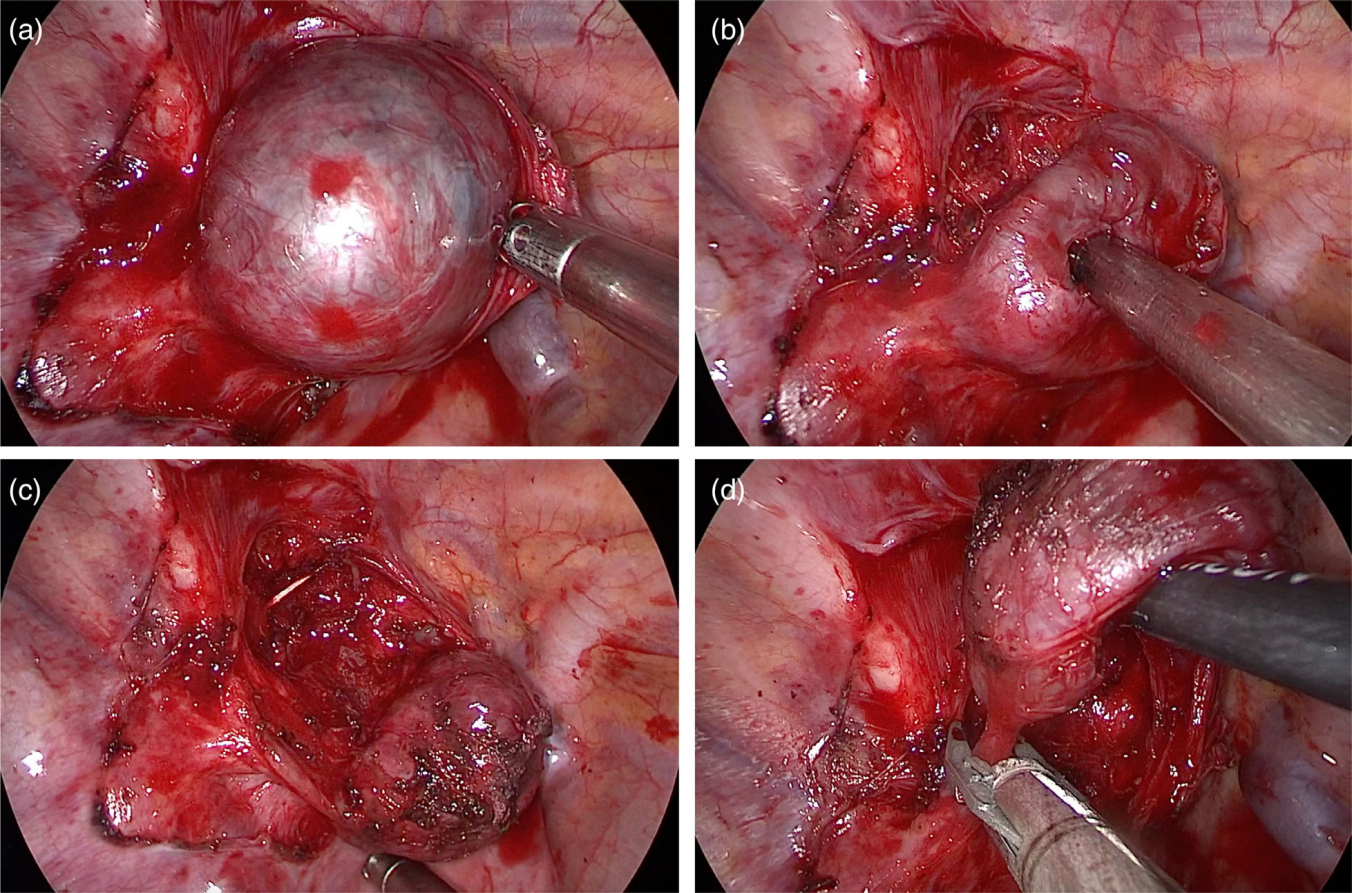
Removal of a malignant schwannoma by video‐assisted thoracoscopic surgery (VATS). The pleura surrounding the tumor was dissected with an energy device (a), the tumor was mobilized exclusively with the use of the energy tool (b). The peripheral attachment of the tumor to the intercostal nerve was identified and then clipped and divided last (c, d).

All patients were placed in the lateral position, under general anesthesia with double‐lumen endotracheal intubation. For tumors that had exclusively intrathoracic growth, a radical surgical resection was carried out as shown in Figure 2. In the case of a mass occupying the spinal canal, the surgery was performed in combination with the assistance of a neurosurgeon.

The VATS technique used at our Thoracic Surgery Unit for the removal of a paravertebral mass consists of an anterior approach with the use of two or three minimally invasive surgeries, of which one of 1 cm at the seventh intercostal space for the insertion of the optic camera (10 mm 30 degrees, Storz), and another of 2–3 cm at the fourth intercostal space as a “utility incision”. In some cases, another 2 cm access is also used through the eighth intercostal space.

In this study, the pleura surrounding the tumor was dissected with an energy device (Harmonic ace+7 or Harmonic 1100; Ethicon) also used to dissect the vessels. The solid and capsulated tumor was mobilized exclusively with the use of the energy tool and then extracted with a specimen retrieval pouch (Endo Catch 10 mm; Medtronic). A cystic lesion was first deflated by aspiration of fluid, mobilized and then completely extracted.^4^

**FIGURE 3:**
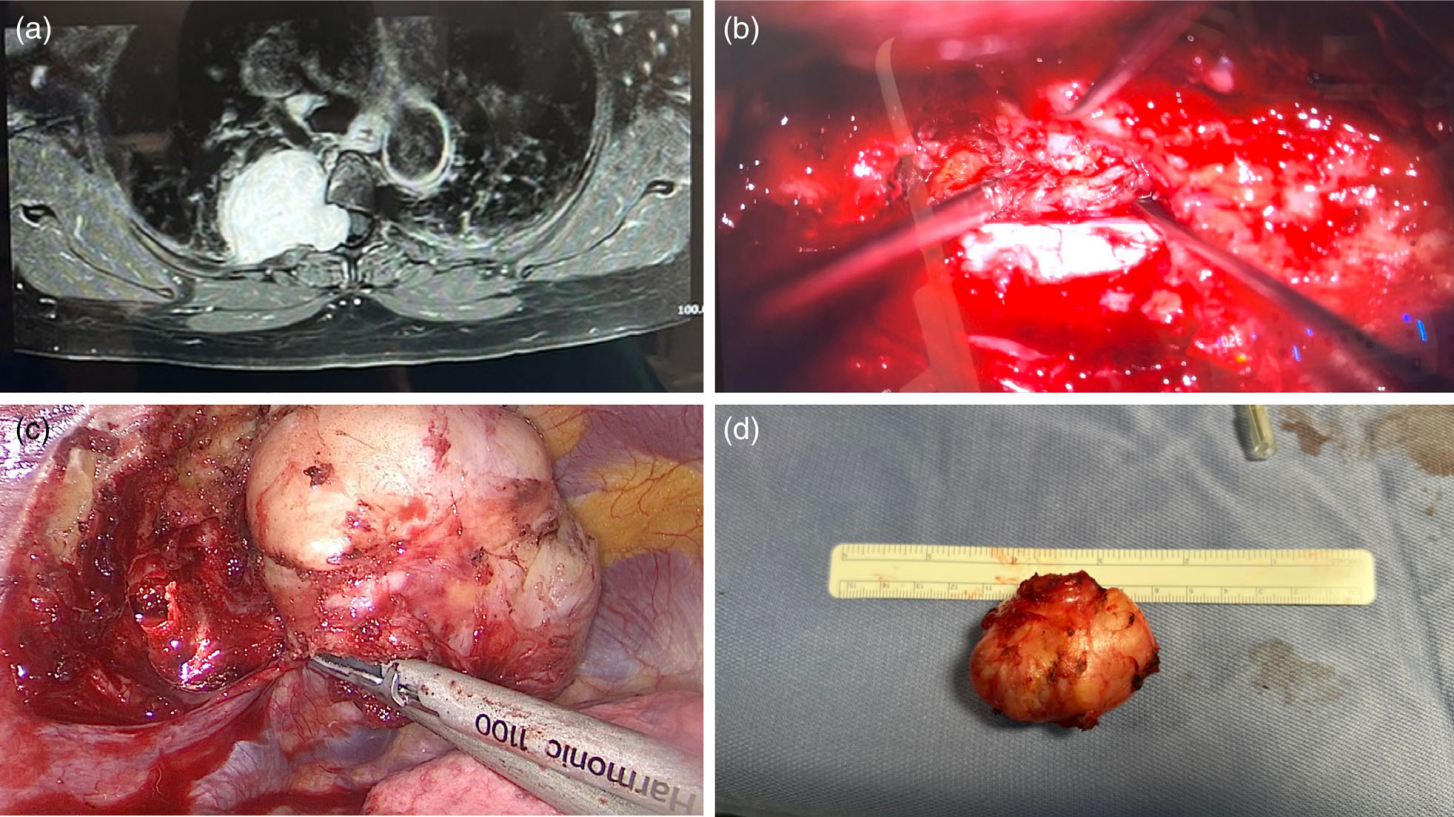
A 44‐year‐old woman with a symptomatic dumbbell tumor located on the right T8/9. (a) The patient underwent combined surgery. The first phase consisted of posterior microneurosurgical removal of spinal component (b) and second phase consisted of thoracoscopic removal of the intrathoracic tumor (c, d).

Combined surgery for dumbbell tumors consisted of a first phase of posterior microneurosurgical removal of spinal component of the tumor carried out by the neurosurgeon and a second phase of thoracoscopic removal of the intrathoracic tumor, as shown in Figure 3. The patient was in a lateral position with a double lumen endotracheal tube already positioned for the neurosurgical element. In this way, after the minimally invasive laminectomy and the fragmentation of the spinal component with the microscope was carried out, the thoracic time of mobilization and removal of the tumor was performed without having to turn the patient from a prone to lateral position.

## RESULTS

A summary of the patient characteristics is given in Table 1. Twenty‐five patients were diagnosed with a paravertebral lesion of which 19 (76%) had solid features and six (24%) had cystic features. Seventeen (68%) were located on the right, while eight (32%) were on the left. The diagnosis of paravertebral lesions was as follows: 18 schwannoma (72%), five neurofibroma (20%), and two malignant schwannoma (8%). The median age was 47 ± 3.5 years and 13 of 25 patients were male. The mean diameter of the lesions was 29 ± 3.8 mm. The mean Hounsfield unit (HU) density at CT scan was 35 ± 16.9. All tumors were in the posterior mediastinum. In four cases (12%), the tumor showed an intraspinal extension. Symptoms were reported in 10 cases (40%) and included cough, dyspnea and neurological symptoms. Comparing clinical cases of solid and cystic neurogenic tumors, no differences in demographic, operational and postoperative characteristics were found, as shown in Table 2. More malignant disease was observed in the group of cystic than solid lesions but further studies are needed to substantiate thess findings. Surgery was only performed by the thoracic surgeon when the lesion did not show an intraspinal extension, while it was conducted in combination with the neurosurgeon who performed laminectomy in patients with dumbbell injury. The average duration of operating times was 113.8 ± 47 min and the mean blood loss was 200 ± 70 mL. The postoperative course was free of intra‐ and postoperative complications in 21 cases (84%). In three cases there was a need for pain relief for postoperative neuropathic pain (12%). Median length of stay was 3 ± 0.7 days. None of the patients had recurrence until 6 months of follow‐up. Surgery was performed using VATS in 72% of cases and in open with traditional thoracotomy in 18%. The measurement of acute postoperative pain using visual analog scales (VAS) was 28.33 ± 10.4 versus 51.43 ± 8.9 between VATS and thoracotomy procedures the discharge of the patients on the postoperative day was, on average, 2.61 ± 0.5 versus 3.51 ± 0.53, respectively (*p*‐value <0.001). The comparison between VATS and thoracotomy procedures is shown in Table 3.

**TABLE 1:** Demographic and operative data

Patients	Age	Sex	FEV1	Side of tumor	Tumor size (mm)	Blood loss (mL)	Operative time (min)	Surgery	Diagnosis	CT scan features	Outcome (POD)	VAS (0–100)	Symptoms	HU density	Complications
1	26	F	90	Right; T2/3	30	200	80	VATS	Malignant schwannoma	Cystic	2	20	Neurological, dyspnea	10	Nil
2	64	F	98	Right; T8/9	26	300	70	VATS	Schwannoma	Solid	3	20		35	Nil
3	55	F	89	Right; T8/9	32	200	60	Open	Schwannoma	Solid	3	40		33	Nil
4	43	M	80	Right; T6/7	25	250	60	Open	Schwannoma	Solid	3	50	Neurological	45	Nil
5	60	F	88	Left; T9/10	33	300	210	VATS	Schwannoma	Solid	3	40		32	Nil
6	44	F	98	Right; T8/9	30	200	200	VATS + laminectomy	Schwannoma	Solid, dumbbell	4	40	Neurologial	42	Neuropathic pain
7	52	M	102	Right; T7/8	26	100	65	VATS	Schwannoma	Solid	3	30		45	Nil
8	55	F	84	Left; T8/9	28	200	80	VATS	Schwannoma	Solid	3	20		36	Nil
9	39	M	80	Left; T8/9	23	400	170	Open + laminectomy	Schwannoma	Solid, dumbbell	4	60	Neurological	50	Nil
10	63	F	100	Right; T9/10	26	200	80	VATS	Schwannoma	Cystic	2	30		−5	Nil
11	54	F	96	Right; T9/10	35	300	150	Open + laminectomy	Schwannoma	Solid, dumbbell	3	60	Neurological	47	Converted to thoracotomy
12	35	M	85	Right; T2/3	27	200	200	VATS	Neurofibroma	Solid	2	20	Sweat	36	Nil
13	47	M	77	Left: T9/10	29	100	90	VATS	Schwannoma	Cystic	3	10		−5	Nil
14	36	F	96	Right; T9/10	30	200	130	VATS	Schwannoma	Solid	3	10		34	Nil
15	64	F	78	Left; T8/9	31	150	120	Open	Schwannoma	Solid	3	60		35	Nil
16	55	M	90	Right; T6/7	26	150	80	Open	Neurofibroma	Solid	4	40		38	Nil
17	47	F	85	Right; T8/9	21	200	90	VATS	Schwannoma	Solid	2	20		33	Nil
18	51	F	101	Right; T6/7	37	200	130	VATS	Malignant schwannoma	Cystic	3	40	Sweat	3	Nil
19	48	M	85	Left; T8/9	33	150	70	VATS	Neurofibroma	Solid	3	30		42	Nil
20	46	M	84	Right; T7/8	31	150	90	VATS	Schwannoma	Solid	2	30		47	Nil
21	39	F	80	Left: T9/10	27	200	140	VATS	Schwannoma	Cystic	2	40	Neurological	11	Nil
22	47	M	98	Right; T9/10	26	250	170	Open + laminectomy	Schwannoma	Solid, dumbbell	4	50	Neurological, dyspnea	35	Neuropathic pain
23	55	M	82	Right; T7/8	30	150	80	VATS	Neurofibroma	Solid	3	40		44	Nil
24	58	F	79	Left; T7/8	29	200	130	VATS	Schwannoma	Cystic	2	30	Neurological	10	Nil
25	49	F	80	Right; T8/9	33	100	100	VATS	Neurofibroma	Solid	3	40		35	Neuropathic pain

Abbreviations: FEV1‐ forced expiratory volume 1; HU, Hounsfield unit; POD, postoperative day; VATS, video‐assisted thoracoscopic surgery.

**TABLE 2:** Clinical cases of solid and cystic neurogenic tumors

Variables	All cases (*n* = 25)	Solid lesion group (*n* = 19)	Cystic lesion group (*n* = 6)	*p‐*value
Age, years (*mean*)	49.3 ± 9.5	49.8 ± 8.4	47.3 ± 13.4	0.58
Sex (male), *n* (%)	10 (40)	9 (47)	1 (17)	0.19
FEV1, (%)	88.2 ± 8.16	88.3 ± 7.5	87.8 ± 10.8	0.89
Symptoms, *n* (%)	10 (40)	6 (32)	4 (67)	0.13
Side of tumor				
Right	17 (68)	14 (74)	3 (50)	0.28
Left	8 (32)	5 (26)	3 (50)	0.28
Tumor size (mm)	28.96 ± 3.76	28.7 ± 3.8	29.6 ± 3.8	0.61
HU density	29.76 ± 16.9	39.16 ± 5.7	4.0 ± 7.5	<0.001
Histology, *n* (%)				
Schwannoma	18 (72)	14 (74)	4 (67)	0.74
Neurofibroma	5 (20)	5 (26)	0 (0)	0.17
Malignant schwannoma	2 (8)	0 (0)	2 (33)	0.01

Abbreviation: FEV1‐ forced expiratory volume 1; HU, Hounsfield unit.

**TABLE 3:** Comparison between VATS and open procedures

Variables	VATS (*n* = 18)	Open (*n* = 7)	*p‐*value
Operative time (mean*)*	113 ± 47	115 ± 49	0.92
Blood loss (mean*)*	186 ± 56	242 ± 88	0.07
POD discharge (mean)	2.61 ± 0.5	3.51 ± 0.53	<0.001
VAS (mean)	28.33 ± 10.4	51.43 ± 8.9	<0.001
Complications *n* (%)	2 (11)	1 (14)	0.83

Abbreviations: POD, postoperative day; VAS, visual analog scale; VATS, video‐assisted thoracoscopic surgery.

## DISCUSSION

Intrathoracic neurogenic tumors are predominantly located in the paravertebral area, less in the chest wall. Other sites of origin of these tumors are extremely rare.^5^ We know that these tumors are often benign in adults and are asymptomatic in most cases and that surgical excision is the treatment of choice.

In 1978, a study conducted by Davidson et al. on the management of intrathoracic neurogenic tumors showed that out of 55 cases of ITNs, 41 were completely asymptomatic. The site of such tumors was 52 in the posterior mediastinum and three on the lateral chest wall.

The histological distribution was 39 neurofibroma/schwannoma, 13 ganglioneuroma and three neuroblastoma. Of the 39 benign nerve tissue tumors, there was a recurrence in one case after 6 years, and this patient subsequently underwent reintervention.^6^

In our study, we found that out of 25 cases of ITNs diagnosed in our thoracic surgery unit, 92% were benign tumors.

The preoperative diagnosis of INTs is challenging and requires definitive post‐surgical histological confirmation. Schwannoma and neurofibroma are the most frequent ITNs in adults and have similar radiological features. On CT scan they appear as round, well‐encased masses. They are of solid density most of the time but can also appear as cystic lesions.

The MRI characteristics of these tumors include an isointense or hypointense T1 signal and heterogeneous hyperintense T2 signal. MRI clearly depicts the margins of these tumors, especially when they grow through the intervertebral foramina. Tumors that have a paravertebral intrathoracic portion or extend into the spinal canal are called hourglass or dumbbell tumors.^7^

The first bibliographical data regarding a minimally invasive surgical approach of ITNs date back to 1992 and was in a study by Landreneau et al. who reported the case of a thoracoscopic resection of a neurogenic tumor of the posterior mediastinum without intraspinal extension.^8^

The approach with minimally invasive surgery is widely accepted as effective in the surgical treatment of intrathoracic neurogenic tumors. This approach is advantageous compared to open surgery because VATS gives a better view of the posterior mediastinum. In addition, there is a reduction in postoperative pain and the consumption of painkillers because there is no rib widening by minimally invasive surgery. In the comparison between VATS and open surgery in the treatment of ITNs it is clear that there is a reduction in postoperative stay days in favor of VATS.^9^ Some authors report a contraindication for minimally invasive surgery of malignant neurogenic tumors, those larger than 6 cm, those that infiltrate the spinal canal or that are localized at the ends of the pleural cavity; that is, at the height of the first and last thoracic vertebrae, in the respective paravertebral areas. We believe that there are no absolute contraindications to minimally invasive surgery because the best vision that is guaranteed by the vision magnified by the optics of thoracoscopy allows better control of the injury to avoid complications such as blood loss.

About 10% of ITNs can have a spinal extension and safe removal of these tumors requires a one‐stage combined neurosurgical and thoracic operation. Vallières et al. in 1995 described the surgical approach by combining posterior microneurosurgical and anterior VATS techniques.

This combined surgical approach appears safe and could become the preferred method for removal of most benign posterior mediastinal dumbbell tumors.^10^

In our study, we found that out of 25 cases of ITNs diagnosed in our thoracic surgery unit, 92% were benign tumors. Considering the radiological characteristics of the posterior mediastinal masses found, 19 (76%) were solid lesions and six (24%) were cystic lesions. The lesions with solid appearance, well capsulated, localized in the paravertebral area together with the anamnestic collection and the objective examination were strongly suggestive of ITNs. In addition to assessing morphological aspects with CT, it is also useful to assess the presence of cleavage plans and possible spinal extension by MRI. In our study, the tumor showed spinal extension in 12% of cases. The surgical removal technique of choice is minimally invasive surgery in most cases of tumors that grow entirely in the paravertebral area, without signs of infiltration to the surrounding structures.

The combined surgery for dumbbell tumors consists of a first phase of posterior microneurosurgical removal of spinal component of tumor carried out by the neurosurgeon and a second phase of thoracoscopic removal of the intrathoracic tumor. Our preference is that neurosurgery is carried out with the patient in a lateral position with a double lumen endotracheal tube already positioned. In this way, after the minimally invasive laminectomy and the fragmentation of the spinal component with microscopy has been carried out, the thoracic time of mobilization and removal of the tumor is carried out without having to turn the patient from the prone to the lateral position. ITNs that appear as cystic lesions on CT scan did not show a spinal extension in our study. Surgical removal in thoracoscopy of the entire lesion can be difficult because it can break the capsule during dissection.^11^ After dissecting the pleura, the contents of the cystic lesion are aspirated to facilitate its mobilization and excision.

In conclusion, we report our experience of managing paravertebral ITNs in which 92% of cases were benign tumors. Such tumors underwent minimally invasive surgical resection, including dumbbell tumors. The cystic density of ITNs is not related to the spinal extension of the tumor and may be expression of malignant tumor. However, this was a retrospective study and further studies are warranted in the future.

## AUTHOR CONTRIBUTIONS

The authors confirm contribution to the paper as follows: study conception and design: Alfonso Fiorelli, Stefano Forte, Gaetana Messina; data collection: Beatrice Leonardi, Rosa Mirra; analysis and interpretation of results: Francesco Leone, Vincenzo Di Filippo, Davide Gerardo Pica; draft manuscript preparation: Francesca Capasso, Mary Bove, Antonio Noro, Giorgia Opromolla, Mario Martone, Sabrina De Angelis. All authors reviewed the results and approved the final version of the manuscript.

## CONFLICT OF INTEREST

The authors confirm that there are no conflicts of interest.
